# Machine learning-based prediction of relapse in rheumatoid arthritis patients using data on ultrasound examination and blood test

**DOI:** 10.1038/s41598-022-11361-y

**Published:** 2022-05-04

**Authors:** Hidemasa Matsuo, Mayumi Kamada, Akari Imamura, Madoka Shimizu, Maiko Inagaki, Yuko Tsuji, Motomu Hashimoto, Masao Tanaka, Hiromu Ito, Yasutomo Fujii

**Affiliations:** 1 Department of Human Health Sciences, Graduate School of Medicine, Kyoto University, 53 Kawahara-cho, Shogoin, Sakyo-ku, Kyoto, 606-8507 Japan; 2 Department of Biomedical Data Intelligence, Graduate School of Medicine, Kyoto University, Kyoto, Japan; 3 Department of Advanced Medicine for Rheumatic Diseases, Graduate School of Medicine, Kyoto University, Kyoto, Japan; 4 Department of Clinical Immunology, Graduate School of Medicine, Osaka City University, Osaka, Japan; 5 Department of Orthopaedic Surgery, Graduate School of Medicine, Kyoto University, Kyoto, Japan; 6 Department of Orthopaedic Surgery, Kurashiki Central Hospital, Okayama, Japan

**Keywords:** Ultrasound, Rheumatoid arthritis, Machine learning

## Abstract

Recent effective therapies enable most rheumatoid arthritis (RA) patients to achieve remission; however, some patients experience relapse. We aimed to predict relapse in RA patients through machine learning (ML) using data on ultrasound (US) examination and blood test. Overall, 210 patients with RA in remission at baseline were dichotomized into remission (n = 150) and relapse (n = 60) based on the disease activity at 2-year follow-up. Three ML classifiers [Logistic Regression, Random Forest, and extreme gradient boosting (XGBoost)] and data on 73 features (14 US examination data, 54 blood test data, and five data on patient information) at baseline were used for predicting relapse. The best performance was obtained using the XGBoost classifier (area under the receiver operator characteristic curve (AUC) = 0.747), compared with Random Forest and Logistic Regression (AUC = 0.719 and 0.701, respectively). In the XGBoost classifier prediction, ten important features, including wrist/metatarsophalangeal superb microvascular imaging scores, were selected using the recursive feature elimination method. The performance was superior to that predicted by researcher-selected features, which are conventional prognostic markers. These results suggest that ML can provide an accurate prediction of relapse in RA patients, and the use of predictive algorithms may facilitate personalized treatment options.

## Introduction

Rheumatoid arthritis (RA), characterized by synovial inflammation that causes progressive joint damage and disability, is among the most frequent chronic inflammatory diseases^1^. Recent effective therapies enable most RA patients to achieve remission; however, some patients experience relapse^2,3^. Several clinical information and biological markers (e.g., gender, disease duration, age, C-reactive protein (CRP), erythrocyte sedimentation rate (ESR), rheumatoid factor (RF)) constitute prognostic factors in RA^4^.

Ultrasound (US) is a non-invasive and sensitive method for detecting inflammatory soft tissue and early bone lesions; therefore, the technique is commonly used to assess RA’s disease activity^5^. Several studies show that detecting synovitis with the US is associated with the disease progression of RA^6–11^. Recently, our group demonstrated the utility of superb microvascular imaging (SMI), a recent innovative type of Doppler US technology used for visualizing minute vessels with low blood flow velocity, in predicting relapse in RA patients^12^. Among joints generally assessed with the US, we showed that wrist and metatarsophalangeal (MTP) SMI scores might be vital for predicting RA relapse, depending on patients’ baseline disease activity^12,13^. However, it is unclear whether combining US data and other features, including clinical and biological markers, improves relapse prediction.

Machine learning (ML) is a type of artificial intelligence that encompasses algorithmic methods that enable machines to solve problems. One of the advantages of ML is the ability to analyze diverse data types (e.g., demographic data, laboratory findings, and imaging data) and incorporate them into prognosis prediction^14^. It can uncover useful patterns of features for prediction that would be difficult or impossible for even well-trained individuals to identify^15^. There are hundreds of studies on the application of ML in autoimmune diseases and ML generally achieved promising predictive results^16,17^. In RA, several studies already show the prediction of prognosis using ML^17–23^. However, to our knowledge, no studies have used US data. This study aims at predicting relapse in patients with RA through ML using data on US examination and blood test. Our novel prediction model may lead to a better assessment of relapse risk and enable personalized treatment for RA patients.

## Results

### Prediction of relapse in RA patients using US examination data, blood test data, and all data

**Figure 1:**
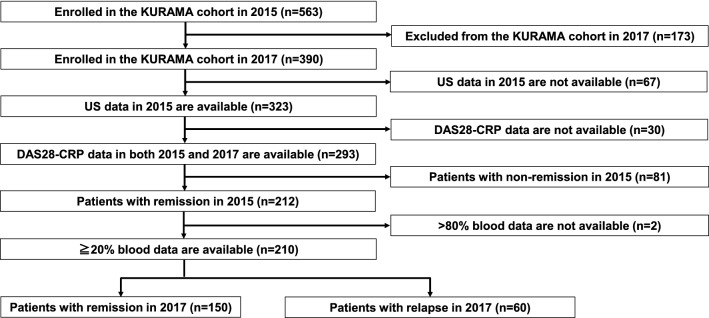
Flow chart depicting patient selection. From 563 RA patients enrolled in the KURAMA cohort in 2015, 390 patients with available follow-up data in 2017 were selected. Next, 323 patients whose US data were available in 2015 were selected. Of the 323 patients, DAS28-CRP data in 2015 and 2017 were available in 293 patients and 81 patients with non-remission (DAS28-CRP ≥ 2.3) in 2015 were excluded. Two of the 212 patients in remission (DAS28-CRP < 2.3) in 2015 lacking the most blood test data (> 80%) were excluded. Finally, the remaining 210 patients were divided into Group 1 (patients with remission in 2017, n = 150) and Group 2 (patients with relapse in 2017, n = 60). *KURAMA* Kyoto University Rheumatoid Arthritis Management Alliance, *US* ultrasound, *DAS28* disease activity score on 28 joints, *CRP* C-reactive protein.

First, we investigated whether a combination of US examination data and blood test data improves the prediction of relapse in RA patients (n = 210) enrolled in the Kyoto University Rheumatoid Arthritis Management Alliance (KURAMA) cohort. A flow chart depicting patient selection is shown in Fig. 1. Characteristics of patients with remission (n = 150) and relapse (n = 60) are shown in Table 1. In patients with relapse, several clinical and biological markers associated with RA disease activity (disease activity score on 28 joints-CRP (DAS28-CRP), simplified disease activity index (SDAI), clinical disease activity index (CDAI), Health Assessment Questionnaire (HAQ), and patient global assessment with visual analog scale (Pt-VAS)) were significantly higher than those in patients with remission. Using 14 data on US examination, 59 data on blood test (including five data on patient information), and all data (US examination data and blood test data), predictive performance was assessed by three ML classifiers [Logistic Regression, Random Forest, and the extreme gradient boosting (XGBoost)]. Supplementary Table 1 shows the detailed list of features. Consequently, in the Random Forest and XGBoost model, area under the receiver operator characteristic curve (AUCs) calculated using all data were higher than those calculated using US or blood test data (Table 2). The highest AUC (0.677) was obtained with Random Forest using all data. These results suggest that a combination of US examination data and blood test data better predicts relapse in RA patients.

**Table 1:** Patient characteristics (n = 210).

	Median (range) or n (%)				*P*-value
	Remission (n = 150)		Relapse (n = 60)		
Age, years	63.8	(28.1–81.4)	66.8	(20.4–91.4)	0.18^#^
Female, n (%)	123	(82.0)	49	(81.7)	1.00^§^
Disease duration, years	7.1	(0.9–71.6)	9.9	(1.2–41.7)	0.06^#^
RF positive, n (%)	111	(74.0)	50	(83.3)	0.20^§^
Anti-CCP positive, n (%)	109	(72.7)	46	(76.7)	0.61^§^
CRP, mg/dl	0.1	(0.0–3.6)	0.1	(0.0–3.6)	0.51^#^
DAS28-CRP	1.3	(1.0–2.3)	1.6	(1.0–2.3)	0.007^#^
SDAI	2.5	(0.0–8.8)	3.5	(0.2–10.4)	0.0009^#^
CDAI	2.2	(0.0–8.8)	3.2	(0.1–9.8)	0.002^#^
HAQ	0.3	(0.0–1.9)	0.5	(0.0–2.5)	0.007^#^
Pt-VAS (mm)	10.0	(0.0–70.0)	13.0	(0.0–80.0)	0.03^#^
Use of glucocorticoid, n (%)	27	(18.0)	15	(25.0)	0.26^§^
Use of methotrexate, n (%)	116	(77.3)	34	(56.7)	0.004^§^
Use of biologics, n (%)	74	(49.3)	26	(43.3)	0.45^§^
TNF inhibitors, n (%)	45	(30.0)	15	(25.0)	0.50^§^
Tocilizumab, n (%)	18	(12.0)	6	(10.0)	0.81^§^
Abatacept, n (%)	10	(6.7)	4	(6.7)	1.00^§^
Tofacitinib, n (%)	1	(0.7)	1	(1.7)	0.49^§^

The groups are defined as follows: Remission: Patients with remission in both 2015 and 2017. Relapse: Patients with remission in 2015 and relapse in 2017.

*RF* rheumatoid factor, *CCP* cyclic citrullinated peptide antibody, *CRP* C-reactive protein, *DAS28* disease activity score on 28 joints, *SDAI* simplified disease activity index, *CDAI* clinical disease activity index, *HAQ* Health Assessment Questionnaire, *Pt-VAS* patient global assessment with visual analog scale. ^#^Mann–Whitney *U* test; ^§^Fisher’s exact test.

**Table 2:** AUCs for predicting relapse in RA patients using US examination data, blood test data, or all data calculated by each model.

Model	AUC		
	US	Blood	All
Logistic Regression	0.659	0.571	0.645
Random Forest	0.621	0.615	0.677
XGBoost	0.650	0.577	0.664

*RA* rheumatoid arthritis, *US* ultrasound, *AUC* area under the curve.

### Prediction of relapse in RA patients using researcher-selected or RFE-selected features

**Figure 2:**
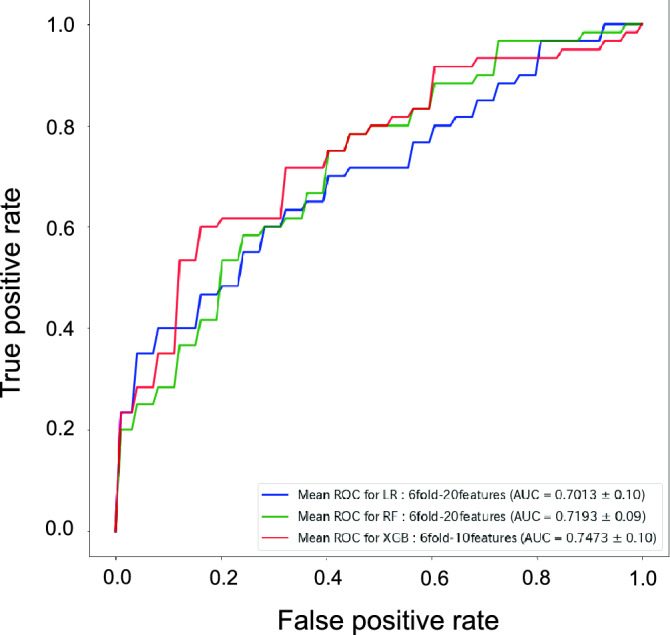
ROC curves for predicting relapse in RA patients. ROC curves of Logistic Regression, Random Forest, and XGBoost for predicting relapse in RA patients are shown. *ROC* receiver operating characteristics, *RA* rheumatoid arthritis.

Next, we applied the recursive feature elimination (RFE) selection algorithm to the prediction to remove weak features and improve the prediction performance. We also selected ten features (gender, disease duration, age, wrist SMI score, MTP SMI score, ESR (1 h), CRP, RF, anti-CCP, and MMP-3) typically associated with disease activity and prognosis in RA patients and compared the results. The best Logistic Regression and Random Forest models utilized 20 and ten RFE-selected features for the best XGBoost model (Supplementary Table 2). RFE-selected features are shown in Supplementary Table 3. AUCs, accuracies, precisions, recalls, and F1-scores were higher in the prediction using RFE-selected features than that using researcher-selected features (Table 3). Among the three ML models, XGBoost showed the highest prediction result (AUC = 0.747, Fig. 2), and the AUC was also higher than the prediction using all data (Tables 2, 3). In the prediction by XGBoost, ten features, including four US examination data, five blood text data, and a piece of patient information were selected (Table 4). These results suggest that RFE-selected features are suitable for prediction in ML, compared with researcher-selected features. XGBoost relapse prediction shows the best performance, and all features (US, blood, and patient information) may be essential.

**Table 3:** Prediction results of relapse in RA patients using researcher/RFE-selected features calculated by each model.

Model	AUC		Accuracy		Precision		Recall		F1-Score	
	Researcher	RFE	Researcher	RFE	Researcher	RFE	Researcher	RFE	Researcher	RFE
Logistic Regression	0.643	0.701	0.629	0.667	0.576	0.626	0.590	0.647	0.571	0.625
Random Forest	0.658	0.719	0.695	0.729	0.619	0.691	0.562	0.595	0.552	0.596
XGBoost	0.590	0.747	0.610	0.776	0.528	0.735	0.532	0.703	0.524	0.706

*RA* rheumatoid arthritis, *RFE* recursive feature elimination, *AUC* area under the curve.

**Table 4:** Comparison of the researcher/RFE-selected features.

Selected by	Features
Researcher	Gender, Disease duration, Age, Wrist SMI score, MTP SMI score, ESR (1 h), CRP, RF, anti-CCP, MMP-3
RFE	Height, Wrist SMI score, MTP SMI score, Lisfranc GS score, Cuneonavicular GS score, LYMPH, ESR (1 h), PLT, ALT, CRE

In RFE-selected features, those selected in XGBoost were shown.

*SMI* superb microvascular imaging, *ESR* erythrocyte sedimentation rate, *CRP* C-reactive protein, *RF* rheumatoid factor, *CCP* cyclic citrullinated peptide antibody, *MMP-3* matrix metalloproteinase-3, *RFE* recursive feature elimination, *MTP* metatarsophalangeal, *LYMPH* lymphocyte count, *PLT* platelet count, *ALT* alanine aminotransferase, *GS* gray scale, *CRE* creatinine.

### The importance of features selected by RFE for predicting relapse in RA patients

**Figure 3:**
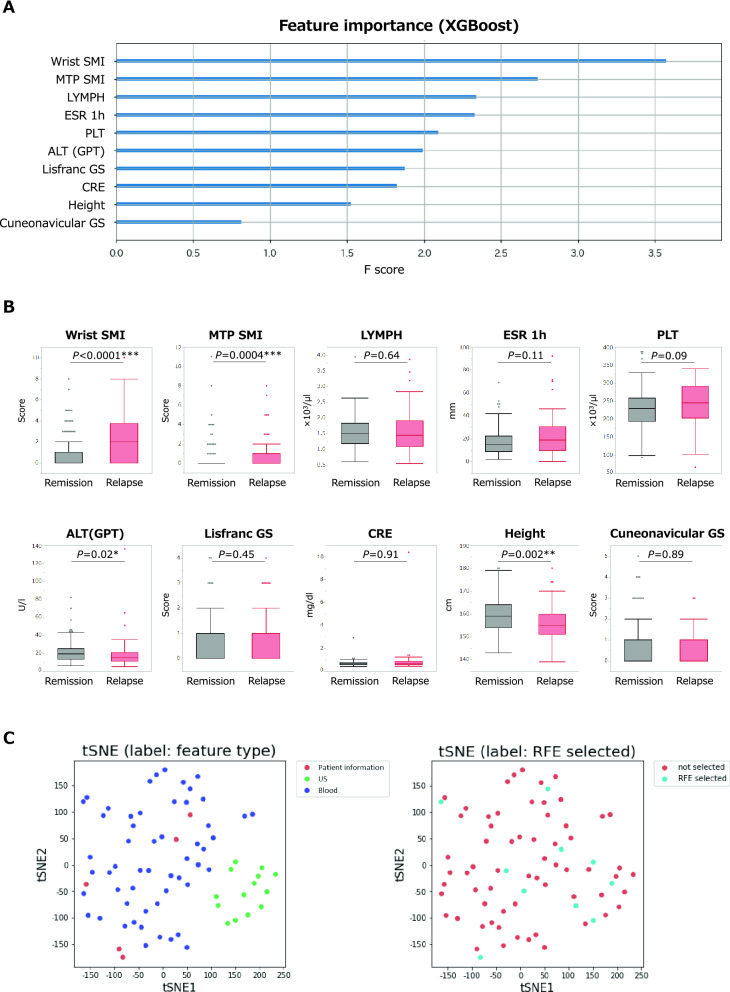
Feature importance for predicting relapse and comparison of each feature between RA patients with remission and relapse. (**A**) Importance of features for predicting relapse calculated by XGBoost model. (**B**) Comparison of each feature between RA patients with remission and relapse. (**C**) Visualization of the characteristics of the selected features in XGBoost model using tSNE. *P < 0.05, **P < 0.01, ***P < 0.001. *SMI* superb microvascular imaging, *MTP* metatarsophalangeal, *LYMPH* lymphocyte count, *ESR* erythrocyte sedimentation rate, *PLT* platelet count, *ALT* alanine aminotransferase, *GPT* glutamic pyruvic transaminase, *GS* gray scale, *CRE* creatinine, *US* ultrasound.

Finally, the importance of features selected by RFE in XGBoost was examined. Wrist and MTP SMI scores were the top two features, followed by four blood test features (lymphocyte count, ESR, platelet count, and alanine aminotransferase) (Fig. 3A). Furthermore, the ten features’ value was compared between patients with remission and relapse (Fig. 3B). Consequently, wrist and MTP SMI scores were significantly higher in patients with relapse. However, alanine aminotransferase (ALT) and height were significantly lower in patients with relapse. There were no significant differences in the remaining six features. The comparison result of all features between patients with remission is shown in Supplementary Table 4. To confirm and visualize the characteristics of the selected features, t-distributed Stochastic Neighbor Embedding (tSNE)^24^ was applied to the standardized input data (Fig. 3C). The RFE selected features were diverse in the embedding space, and it implies the prediction of relapse is made by combining SMI scores with various features. These results suggest that all type of features, especially US data, are important for predicting relapse in RA patients. In addition to the features with significant differences between patients with remission and relapse, those with no significant differences may also contribute to the prediction.

## Discussion

We studied relapse prediction in RA patients through ML using data on US examination and blood test. A combination of US examination and blood test data showed higher AUCs than those calculated using individual data. The result is not surprising because the input of more features generally improves prediction. Next, we used RFE to remove weak features and improve the prediction performance. The prediction using RFE-selected features showed higher performance than that using researcher-selected features, although the number of selected features was the same. The result suggests that RFE uncovered an optimal combination of features for better prediction. Among the ten features selected by RFE in XGBoost, wrist and MTP SMI scores were the top two vital features, suggesting that US data significantly improved prediction of relapse in RA patients. Three features (wrist and MTP SMI scores and ESR) were also included in the researcher-selected features. Wrist and MTP SMI scores were reported as prognostic factors^12,13^, and ESR is one of RA’s most fundamental inflammation markers^1,4^. In the remaining seven features, ALT and height were significantly lower in patients with relapse. ALT is not well-characterized as a prognostic factor, but the elevation is a marker of liver toxicity in RA treatment^25^. There is a possibility that patients with lower ALT may receive lower-intensity therapies, contributing to higher relapse risk. Height is also uncommon as a prognostic factor in RA; however, there is a study that adult height is inversely associated with disease activity^26^, which is compatible with the result. There were no significant differences in six features between patients with remission and relapse. The comparison is a univariate analysis of the total cohort. Therefore, information on the association among features and prognostic significance in patient subgroups is lacking. Further studies on the importance of these features, including underlying biological mechanisms, are required. Among 63 features which were not selected by RFE in XGBoost, several features also had statistical difference in the value between patients with remission and relapse (Supplementary Table 4). This raises the possibility that other relative features with more importance are alternatively selected by RFE.

Among the three ML models, XGBoost, a scalable, distributed gradient-boosted decision tree ML library, achieved the best performance (AUC = 0.747). The model has gained much attention recently due to its superior performance^27,28^, which is compatible with the prediction results in this study. Because the decision tree-based model is adequate for data sets containing various features, Random Forest and XGBoost showed more accuracy than Logistic Regression for mixed data. XGBoost algorithm selects one feature when there is a high correlation between variables, whereas Random Forest randomly selects a feature and learns the correlations of different features across the model. Therefore, XGBoost was considered more accurate in feature selection because it could select a smaller number and more efficient features. In our previous study analyzing almost the same cohort without using ML, the highest AUC was 0.67 for predicting relapse^12^, suggesting that ML using US examination and blood test data improved prediction results. This study’s sample size (n = 210) is typical among previous studies on ML applications to autoimmune diseases^17^. However, larger sample size could improve prediction. In this study, the follow-up period was 2 years, and the results may vary according to follow-up duration. Therefore, the results should be validated in studies conducted in larger populations with multiple follow-up times. Recent studies showed the possible application of ML to the measurement of US/X-ray images^29,30^. A combination of such technologies and our ML model can be a promising approach for convenient and better prediction of relapse.

In conclusion, we established an improved model for predicting relapse in RA patients through ML. The combination of data on US examination and blood test was a unique approach of this study, and US data were shown to be essential for prediction. The findings may lead to a better assessment of relapse risk and enable the selection of personalized treatment strategies for RA patients.

## Methods

### Patients

From 563 RA patients enrolled in the KURAMA cohort in 2015, 390 patients with available follow-up data in 2017 were selected (Fig. 1). Next, 323 patients whose US data were available in 2015 were selected. Of the 323 patients, DAS28-CRP data in 2015 and 2017 were available in 293 patients, and 81 patients with non-remission (DAS28-CRP ≥ 2.3) in 2015 were excluded. Two of the 212 patients in remission (DAS28-CRP < 2.3) in 2015 lacking the most blood test data (> 80%) were excluded. Subsequently, the remaining 210 patients were divided into “Remission” (patients with remission in 2017, n = 150) and “Relapse” (patients with relapse in 2017, n = 60).

### Data collection

The US was examined using an Aplio500 (Canon Medical Systems) fitted with a 12 MHz linear probe (18L7). Bilateral joints (second through fifth metacarpophalangeal (MCP), radial wrist, ulnar wrist, second through fifth MTP, Lisfranc, cuneonavicular, Chopart, and ankle) were examined as described previously^31^. The scanning technique and interpretation of lesions were based on Outcomes Measures in Rheumatology (OMERACT)^32^. The former of the two SMI modes (color-coded and monochrome SMI) was used for this study. Regions of interest for SMI were fixed at the same size and depth for each joint type. Under the established four-point scale (0–3) semi-quantitative scoring system^33^, gray scale (GS) and SMI scores were determined on-site by at least two of five sonographers with 1–9 years of experience, and agreement was obtained in weekly meetings attended by all five sonographers. The scores for each group of joints were summed as follows: MCP, bilateral second through fifth MCP; wrist, bilateral radial, and ulnar joints; MTP, bilateral second through fifth MTP; Lisfranc, bilateral Lisfranc joints; Cuneonavicular, bilateral Cuneonavicular joints; Chopart, bilateral Chopart joints; ankle, bilateral ankle joints. There were no missing data in the US examination.

Patient information and blood test data in 2015 were also collected from the KURAMA cohort. Supplementary Table 1 shows the list of features. Cases with more than 80% of the missing features were eliminated. Missing values were complemented with each feature’s median value. For replacing missing values on patients’ height and weight, median values were calculated by gender. In total, 14 US examination data, 54 blood test data, and five data on patient information were available for analysis. For convenience, data on patient information were included in blood test data in this study.

### Prediction models

Three ML classifiers (Logistic Regression, Random Forest, and XGBoost) were employed to predict RA patients’ relapse. The logistic regression model is a generalized linear model and traditional approach for binary classification on clinical prediction. Random Forest is an ensemble algorithm that combines multiple decision trees to build a robust model^34^. It is widely used because of its high interpretability of prediction results. XGBoost is also a decision tree-based ensemble algorithm and achieves more accurate prediction utilizing gradient boosting^35^.

Predictive performance was assessed using the mean AUC by nested stratified six-fold cross-validation (CV). The inner loop, consisting of a three-fold CV, was used to select hyper-parameters by grid-search. The class balance option was set for all models to deal with imbalanced data.

For feature selection, we employed RFE, a method for extracting subsets of features that contribute to prediction performance by recursive processing. Since RFE allows us to set the size of the final feature subset, we varied the value within [5, 10, 20, 30, 50], finally selecting the number of features that showed the best AUC. Analyses and model constructions were performed with Python 3.8 packages (Scikit-learn 0.23 and XGBoost 1.1.1).

### Ethical statements

This study was conducted following the principles set down in the Declaration of Helsinki and was approved by the ethics committee of Kyoto University (R0357). All patients provided written informed consent.

## Supplementary Information

Supplementary Tables.
