# Supplementary material for: Machine learning-based prediction of relapse in rheumatoid arthritis patients using data on ultrasound examination and blood test
Source: Sci Rep. 2022 May 4;12:7224. doi: 10.1038/s41598-022-11361-y (PMC9068780; doi:10.1038/s41598-022-11361-y)
Supplement: Supplementary file 1 — Supplementary Tables. [file 41598_2022_11361_MOESM1_ESM.docx]

**Supplementary Table 1. List of the features used for machine learning.**

| Data type | Features | Abbreviations  (if applicable) |
| --- | --- | --- |
| Patients' information | Age |  |
|  | Gender |  |
|  | Height |  |
|  | Weight |  |
|  | Disease duration |  |
| Ultrasound examination | Gray scale score in the metacarpophalangeal joints | MCP GS score |
|  | Superb microvascular imaging score in the metacarpophalangeal joints | MCP SMI score |
|  | Gray scale score in the wrist joints | Wrist GS score |
|  | Superb microvascular imaging score in the wrist joints | Wrist SMI score |
|  | Gray scale score in the metatarsophalangeal joints | MTP GS score |
|  | Superb microvascular imaging score in the metatarsophalangeal joints | MTP SMI score |
|  | Gray scale score in the Lisfranc joints | Lisfranc GS score |
|  | Superb microvascular imaging score in the Lisfranc | Lisfranc SMI score |
|  | Gray scale score in the Cuneonavicular joints | Cuneonavicular GS score |
|  | Superb microvascular imaging score in the Cuneonavicular joints | Cuneonavicular SMI score |
|  | Gray scale score in the Chopart joints | Chopart GS score |
|  | Superb microvascular imaging score in the Chopart joints | Chopart SMI score |
|  | Gray scale score in the ankle joints | Ankle GS score |
|  | Superb microvascular imaging score in the ankle joints | Ankle SMI score |
| Blood test | Hematocrit | HCT |
|  | Hemoglobin | HGB |
|  | Red blood cell count | RBC |
|  | Mean corpuscular volume | MCV |
|  | Mean corpuscular hemoglobin | MCH |
|  | Mean corpuscular hemoglobin concentration | MCHC |
|  | White blood cell count | WBC |
|  | Platelet count | PLT |
|  | Neutrophil count | NEUT |
|  | Lymphocyte count | LYMPH |
|  | Neutrophil (%) |  |
|  | Lymphocyte (%) |  |
|  | Monocyte (%) |  |
|  | Eosinophil (%) |  |
|  | Basophil (%) |  |
|  | 1-hour-value of the erythrocyte sedimentation rate | ESR (1h) |
|  | 2-hour-value of the erythrocyte sedimentation rate | ESR (2h) |
|  | C-reactive protein | CRP |
|  | Rheumatoid factor | RF |
|  | Alanine aminotransferase | ALT |
|  | Lactate dehydrogenase | LDH |
|  | Cholinesterase | Ch-E |
|  | Blood urea nitrogen | BUN |
|  | Amylase | AMY |
|  | Aspartate aminotransferase | AST |
|  | Alkaline phosphatase | ALP |
|  | Total bilirubin | T-Bil |
|  | Triglyceride | TG |
|  | Total protein | TP |
|  | Potassium | K |
|  | Chloride | Cl |
|  | Albumin | ALB |
|  | Calcium | Ca |
|  | Chloride | IP |
|  | Creatinine | CRE |
|  | Estimated glomerular filtration rate | eGFR |
|  | Leucine aminopeptidase | LAP |
|  | γ-glutamyl transpeptidase | γ-GTP |
|  | Total cholesterol | T-CHO |
|  | High-density lipoprotein cholesterol | HDL-CHO |
|  | Low-density lipoprotein cholesterol | LDL-CHO |
|  | Fetal hemoglobin | HbF |
|  | Hemoglobin A1c | HbA1c |
|  | Labile hemoglobin A1c (#C) fraction | #C |
|  | Creatine Kinase | CK |
|  | Sodium | Na |
|  | Glucose | Glu |
|  | Krebs von den Lungen-6 | KL-6 |
|  | Bone-type alkaline phosphatase | Bone-type ALP |
|  | Total homocysteine | tHcy |
|  | Anti-cyclic citrullinated peptide antibody | anti-CCP |
|  | Undercarboxylated osteocalcin | ucOC |
|  | Tartrate-resistant acid phosphatase 5b | TRACP-5b |
|  | Matrix metalloproteinase-3 | MMP-3 |

**Supplementary Table 2. AUCs for each feature set in each model.**

|  | Researcher | RFE | | | | | All | Blood | US |
| --- | --- | --- | --- | --- | --- | --- | --- | --- | --- |
|  |  | 5-features | 10-features | 20-features | 30-features | 50-features |  |  |  |
| Logistic Regression | 0.643 | 0.671 | 0.689 | **0.701** | 0.671 | 0.673 | 0.645 | 0.571 | 0.659 |
| Random Forest | 0.658 | 0.679 | 0.667 | **0.719** | 0.708 | 0.694 | 0.677 | 0.615 | 0.621 |
| XGBoost | 0.590 | 0.740 | **0.747** | 0.702 | 0.690 | 0.677 | 0.664 | 0.577 | 0.650 |

Abbreviations: AUC, area under the curve; RFE, recursive feature elimination; US, ultrasound.

The highest AUCs for each model are shown in bold.

**Supplementary Table 3. RFE-selected features for the best AUC model.**

| 20-features for Logistic Regression | 20-features for Random Forest | 10-features for XGBoost |
| --- | --- | --- |
| Weight | Age | Height |
| Wrist SMI score | Height | Wrist SMI score |
| MTP GS score | Disease duration | MTP SMI score |
| MTP SMI score | Wrist SMI score | Lisfranc GS score |
| Lisfranc GS score | MCH | Cuneonavicular GS score |
| Lisfranc SMI score | WBC | PLT |
| Cuneonavicular SMI score | PLT | LYMPH |
| Chopart GS score | Neutrophil | ESR (1h) |
| Ankle SMI score | Lymphocyte | ALT |
| HCT | Monocyte | CRE |
| HGB | LYMPH |  |
| RBC | NEUT |  |
| LYMPH | ESR (1h) |  |
| ESR (1h) | ESR (2h) |  |
| ESR (2h) | RF |  |
| BUN | Ch-E |  |
| IP | TG |  |
| CRE | T-CHO |  |
| HbF | CK |  |
| HbA1c | KL-6 |  |

Abbreviations: See supplementary Table1.

**Supplementary Table 4. Comparison of all features between RA patients with remission and relapse.**

|  | Median value | |  |  |
| --- | --- | --- | --- | --- |
| Features | Remission | Relapse | *P*-value | |
| Age (year) | 63.8 | 66.8 | 0.18 | # |
| Female (%) | 82.0 | 81.7 | 1.00 | § |
| Height (cm) | 159.0 | 155.0 | 0.0024 | # |
| Weight (kg) | 55.0 | 53.0 | 0.045 | # |
| Disease duration (year) | 7.1 | 9.9 | 0.060 | # |
| MCP GS score | 1 | 2 | 0.044 | # |
| MCP SMI score | 0 | 0 | 0.025 | # |
| Wrist GS score | 2 | 3 | 0.0019 | # |
| Wrist SMI score | 0 | 2 | <0.0001 | # |
| MTP GS score | 6 | 5 | 0.29 | # |
| MTP SMI score | 0 | 0 | 0.0004 | # |
| Lisfranc GS score | 0 | 0 | 0.45 | # |
| Lisfranc SMI score | 0 | 0 | 0.010 | # |
| Cuneonavicular GS score | 0 | 0 | 0.89 | # |
| Cuneonavicular SMI score | 0 | 0 | 0.31 | # |
| Chopart GS score | 2 | 2 | 0.15 | # |
| Chopart SMI score | 0 | 0 | 0.72 | # |
| Ankle GS score | 2 | 2 | 0.62 | # |
| Ankle SMI score | 0 | 0 | 0.25 | # |
| HCT (%) | 38.9 | 38.6 | 0.81 | # |
| HGB (g/dL) | 12.8 | 12.8 | 0.68 | # |
| RBC (x10^6^/µL) | 4.2 | 4.2 | 0.60 | # |
| MCV (fL) | 94.1 | 92.6 | 0.19 | # |
| MCH (pg) | 31.1 | 30.2 | 0.046 | # |
| MCHC (g/dL) | 33.2 | 32.8 | 0.020 | # |
| WBC (x10^3^/µL) | 5.0 | 5.6 | 0.18 | # |
| PLT (x10^3^/µL) | 229.5 | 245.0 | 0.093 | # |
| NEUT (x10^3^/µL) | 2.9 | 3.4 | 0.16 | # |
| LYMPH (x10^3^/µL) | 1.5 | 1.4 | 0.64 | # |
| Neutrophil (%) | 60.1 | 59.4 | 0.38 | # |
| Lymphocyte (%) | 29.8 | 28.9 | 0.26 | # |
| Monocyte (%) | 6.4 | 5.8 | 0.093 | # |
| Eosinophil (%) | 2.3 | 1.9 | 0.19 | # |
| Basophil (%) | 0.6 | 0.6 | 0.30 | # |
| ESR 1h (mm) | 15.0 | 19.0 | 0.11 | # |
| ESR 2h (mm) | 33.0 | 43.5 | 0.13 | # |
| CRP (mg/dL) | 0.1 | 0.1 | 0.51 | # |
| RF (IU/mL) | 35.3 | 50.1 | 0.075 | # |
| ALT (U/L) | 19.0 | 15.5 | 0.021 | # |
| LDH (U/L) | 190.0 | 186.0 | 0.86 | # |
| Ch-E (U/L) | 295.0 | 271.0 | 0.12 | # |
| BUN (mg/dL) | 14.0 | 17.0 | 0.053 | # |
| AMY (U/L) | 80.0 | 86.0 | 0.26 | # |
| AST (U/L) | 22.0 | 21.0 | 0.25 | # |
| ALP (U/L) | 207.5 | 196.5 | 0.51 | # |
| T-Bil (mg/dL) | 0.7 | 0.7 | 0.20 | # |
| TG (mg/dL) | 98.0 | 84.0 | 0.013 | # |
| TP (g/dL) | 6.9 | 7.1 | 0.59 | # |
| K (mEq/L) | 4.1 | 4.1 | 0.89 | # |
| Cl (mEq/L) | 104.0 | 104.0 | 0.54 | # |
| ALB (g/dL) | 4.1 | 4.0 | 0.10 | # |
| Ca (mg/dL) | 8.9 | 8.9 | 0.69 | # |
| IP (mg/dL) | 3.5 | 3.5 | 0.28 | # |
| CRE (mg/dL) | 0.7 | 0.6 | 0.91 | # |
| eGFR (mL/min/1.73m^2^) | 74.2 | 74.5 | 0.95 | # |
| LAP (U/L) | 49.0 | 49.0 | 0.98 | # |
| γ-GTP (U/L) | 20.0 | 16.5 | 0.080 | # |
| T-CHO (mg/dL) | 202.0 | 190.0 | 0.065 | # |
| HDL-CHO (mg/dL) | 67.0 | 68.0 | 0.62 | # |
| LDL-CHO (mg/dL) | 117.0 | 109.0 | 0.076 | # |
| HbF (%) | 0.3 | 0.3 | 0.035 | # |
| HbA1c (%) | 5.6 | 5.5 | 0.26 | # |
| #C (%) | 1.7 | 1.7 | 0.67 | # |
| CK (U/L) | 85.0 | 69.0 | 0.069 | # |
| Na (mEq/L) | 140.0 | 140.0 | 0.26 | # |
| Glu (mg/dL) | 92.0 | 94.0 | 0.41 | # |
| KL-6 (U/mL) | 256.0 | 238.0 | 0.16 | # |
| Bone-type ALP (µg/L) | 11.9 | 11.2 | 0.46 | # |
| tHcy (nmol/mL) | 9.7 | 10.1 | 0.24 | # |
| anti-CCP (U/mL) | 52.8 | 64.0 | 0.58 | # |
| ucOC (ng/mL) | 3.2 | 2.9 | 0.31 | # |
| TRACP-5b (mU/dL) | 335.5 | 395.0 | 0.42 | # |
| MMP-3 (ng/mL) | 48.2 | 70.7 | 0.0064 | # |
| Abbreviations: See supplementary Table1. #, Mann–Whitney U test; §, Fisher’s exact test. | | | | |
|  |  |  |  |  |
